# Plant strategies for maximizing growth during water stress and subsequent recovery in *Solanum melongena* L. (eggplant)

**DOI:** 10.1371/journal.pone.0256342

**Published:** 2021-09-01

**Authors:** Evelyn F. Delfin, Sarah Tepler Drobnitch, Louise H. Comas

**Affiliations:** 1 Institute of Plant Breeding, College of Agriculture and Food Science, University of the Philippines at Los Baños, Laguna, Philippines; 2 Soil and Crop Department, Colorado State University, Fort Collins, Colorado, United States of America; 3 United States Department of Agriculture, Agricultural Research Service, Fort Collins, Colorado, United States of America; National Research Council of Italy (CNR), ITALY

## Abstract

Climate change is projected to increase the incidence of severe drought in many regions, potentially requiring selection for different traits in crop species to maintain productivity under water stress. In this study, we identified a suite of hydraulic traits associated with high productivity under water stress in four genotypes of *S*. *melongena* L. We also assessed the potential for recovery of this suite of traits from drought stress after re-watering. We observed that two genotypes, PHL 4841 and PHL 2778, quickly grew into large plants with smaller, thicker leaves and increasingly poor hydraulic status (a water-spender strategy), whereas PHL 2789 and Mara maintained safer water status and larger leaves but sacrificed large gains in biomass (a water-saver strategy). The best performing genotype under water stress, PHL 2778, additionally showed a significant increase in root biomass allocation relative to other genotypes. Biomass traits of all genotypes were negatively impacted by water deficit and remained impaired after a week of recovery; however, physiological traits such as electron transport capacity of photosystem II, and proportional allocation to root biomass and fine root length, and leaf area recovered after one week, indicating a strong capacity for eggplant to rebound from short-term deficits via recovery of physiological activity and allocation to resource acquiring tissues. These traits should be considered in selection and breeding of eggplant hybrids for future agricultural outlooks.

## Introduction

Water shortages are responsible for the greatest crop losses around the world and expected to worsen in many regions where agricultural water availability is challenged with precipitation declines due to climatic changes, and/or competition with municipal and industrial needs [1, 2]. To improve crop production under water limitations we need a foundational understanding of traits contributing to crop performance and crop strategies that maintain productivity [3–6]. Furthermore, although traits that drive crop yields alter performance late in plant development, they may be correlated with trait responses to water limitations at earlier growth stages [7].

Many plant processes respond concurrently when plants are under water limitations. However it has been clearly observed that even within the same species, different traits may support or damage crop productivity under water stress [8, 9]. Additionally, the timing and duration of water stress can activate differing trait responses [8, 9]. Despite this, strategies are considered to fall into two broad categories: traits that conserve water (water savers) versus traits that exacerbate declining hydraulic function (water spenders) [10]. In wild ecosystems, both strategies can result in increased survival and reproductive success; “drought tolerance” involves the expression of water saver traits to withstand water deficits until rain events, whereas “drought avoidance” is typical of water spenders that grow and reproduce rapidly before the onset of drought [11–14]. Given that survival alone is a poor benchmark for crops, desirable crop genotypes for drought-prone systems must also maintain high productivity under water limitations [3, 5, 15, 16]. While water savers may effectively limit water loss by closing stomata [17, 18] and engaging in gas exchange only briefly in the morning and at night, this may result in the undesirable reduction of photosynthesis, growth rates, and yield [19–22]. Furthermore, species that cope with low tissue water potential by maintaining turgor through osmotic adjustment, increased cell elasticity, reduced cell size, and protoplasmic resistance often expend energy that reduces productivity [23, 24]. In some species, fast growing water spenders manage to maximize productivity without protecting tissue turgor or hydraulic safety (as do “early vigor” varieties in many species) [25, 26]. In these varieties, the same traits supporting optimal growth under well-watered conditions may also support optimal growth under water deficit [27]. Water spenders with the potential to “avoid” drought and still produce a viable crop may do so by increasing root depth or developing a more efficient root system for water foraging [24, 28–30]. The growth of a deep and extensive root system can come at the expense of larger shoot systems and high yields [31, 32]. Either way, the importance of root traits to crop performance under water stress has been evident; greater root length density can produce higher yields under drought [33].

The ability to recover from damage sustained during drought events varies substantially between species and cultivars and is as important for maintaining total seasonal productivity as traits that minimize damage during drought stress [34]. Some drought tolerance strategies can actually slow post-watering recovery; for example, roots exposed to dry soil can become suberized and limit water uptake until new root growth occurs [35, 36]. Key post-drought recovery traits seem to be focused on rapid restoration of photosynthesis (membrane repair, photosystem protection and repair, pigment protection and repair), hydraulic conductance (aquaporin up-regulation, cavitation reversal, rapid osmotic adjustments), and fine root flushing [37–40].

In the Philippines and much of southeast Asia, climate change is projected to increase the incidence and severity of typhoons but also the intensity of El Niño events, raising the occurrence of severe drought in a historically wet tropical region [41–43]. Due to their high water content, drought disproportionally impacts vegetable crops (as opposed to grains and tree fruits), which form a large component of Philippine agriculture [44]. A major strategy for weathering drought events is selection and cultivation of drought resistant genotypes and germplasm. International institutions such as the International Maize and Wheat Improvement Center (CIMMYT), the World Vegetable Center, the International Crops Institute for Semi-Arid Tropics (ICRISAT) and International Institute of Tropical Agriculture (IITA) have undertaken collections of diverse crop germplasm [45–48]. These groups have successfully screened many crop species, such as wheat and maize, for drought resistant genotypes [49]. Genotypic variability of drought tolerance has been established in nearly all crop species, from wheat, faba bean, and cotton [20, 50, 51] to watermelon [52]. The eggplant, *Solanum melongena* L., is ranked as one of the most important vegetables consumed and produced in the Philippines with an estimated production of 179,000 metric tons worth ~ US$32 million [53, 54]. *S*. *melongena* has been shown to be adversely affected by drought in terms of plant height, total dry weight and fruit yield [55–58]. Reduction in transpiration rate, stomatal conductance and photosynthetic rate of eggplant were also observed upon exposure to different drought stress durations [59]. Preliminary work with Philippine *S*. *melongena* genotypes showed a reduction of 21–29% in fruit production exposed to short-term drought stress [60].

In this study, we select four eggplant genotypes with a range of productivity under short duration water stress, including a reference variety [60]. Previous work has addressed drought- mediated shifts in photosynthetic pigments and reactive oxygen scavengers [57, 61] whereas we focus on traits that reflect the hydraulic functioning of *S*. *melongena*. We measured plant hydraulic, gas exchange, and morphological traits and identified correlations with biomass productivity under a two-week drought and subsequent one-week recovery period. We ask whether there is a tradeoff between water spender or saver strategies and vegetative biomass accumulation during the drought or recovery phase among the four genotypes.

## Methods

### Greenhouse study

A greenhouse experiment was set up to identify physiological traits associated with drought tolerance in eggplant. *Solanum melongena* genotypes PHL 4841, PHL 2778 and PHL 2789 were chosen based on drought performance in previous field and greenhouse trials [60] of 100 germplasm accessions from the National Plant Genetic Resources Laboratory in the Institute of Plant Breeding, University of the Philippines at Los Baños, Laguna, Philippines. *S*. *melongena* ‘Mara’, a released variety from the Institute of Plant Breeding, UP Los Baños was included as a reference variety. Seeds were sown into seedling trays containing fritted clay (Turface Greens Grade, Profile Products, Buffalo Grove, IL, USA) at the end of February in a greenhouse in Fort Collins, CO, USA. After 17–21 days, individuals were transplanted into 7.57 L plastic pots containing 10 kg fritted clay and watered to holding capacity via a drip irrigation before treatments were established. Pots were positioned on two greenhouse benches in a randomized complete block design of two factors: water availability (drought vs. well-watered control) and genotype (four genotypes). There were 5 replicates of each block (40 plants). This basic block design was doubled, and plants were harvested at 2 time points (post-drought and post-recovery) for a total of 80 plants.

Plants were maintained under a combination of natural sunlight and supplemental LED illumination on a 14:10 hour day:night cycle, corresponding to average temperatures of 22 and 29°C. Plants were fertigated using Grow More water soluble fertilizer (Grow More, Inc., Gardena, CA) amended with additional N in the form of urea and additional K in the form of KH_2_PO_4_ to achieve 79.5–22.5–5 ppm N:P:K daily for the first month after transplantation and transitioned to 60-30-120 ppm N:P:K for the remainder of the experiment.

Drought treatments began at 5 weeks after transplanting and lasted for 2 weeks for all blocks (80 plants). “Drought” plants received 30% of evapotranspired water (ET) of “control” plants daily for the first week, and 10% daily in the second week. “Control” plants were given 100% of ET daily. ET was calculated by weighing control pots daily at 14:00 hr to measure evaporative and transpiration water loss relative to 100% holding capacity. After the conclusion of the drought treatment, all remaining plants were re-watered to pot holding capacity.

#### Physiological measurements

Sampling of drought and control plants were carried out from the 11th through the 15th and final day of the drought treatment on 50% of the experimental plant population. To avoid confounding genotype-specific traits with increased stress to experimental plants at the end of the sampling period, one replicate of each genotype was sampled each day. On each day, the third fully expanded leaf of each plant was measured for chlorophyll fluorescence (F_v_/F_m_) from 07:30 until 08:30 using a portable OS5P fluorometer (Opti-Sciences Inc., NH, USA). Each leaf was dark acclimated with leaf clips for 20 minutes prior to measurement. From 09:00 to 12:00 hrs, the same leaves were measured for photosynthetic rate (A_n_), stomatal conductance (*g*_*s*_), and transpiration using the Li-COR 6400XT infrared gas analyzer with attached leaf measurement chamber (LiCOR Inc., Lincoln, Nebraska). Conditions in the leaf measurement chamber were the following: PAR (photosynthetically active radiation) of 1800 μmol m^-2^s^-1^, leaf temperature of 25°C, and CO_2_ concentration of 400 μmol mol^-1^. Instantaneous water use efficiency (WUEi) was calculated as the ratio of A_n_ to g_s_.

Leaf water potential (Ψ_L_) was determined with use of a Scholander pressure chamber (Soil Moisture Equipment Corp., Santa Barbara, CA, USA). The same leaf used for gas exchange measurements was cut from each plant and immediately placed in a plastic bag in a cooler until Ψ_L_ could be measured (up to 1 hour).

After one week under full watering, “recovered” and control plants were again measured as above for chlorophyll fluorescence and leaf water potential.

#### Plant growth measurements

Following physiological measurements, the aboveground portions of drought and control plants were cut and partitioned into leaves and stem. Total leaf area was measured for each plant using a Li-3100C leaf area meter (LiCOR Inc., USA). Partitioned shoot tissue was then oven dried at 60°C for 48 hours and weighed.

The belowground biomass of each plant was washed free of fritted clay and partitioned into fine and coarse roots. A representative sample of fine roots was obtained for each sample and stored in 30% ethyl alcohol for root scanning. Preserved fine roots were scanned in water in 2-D transparency mode with a desktop scanner (EpsonV750, Epson America Inc., USA) and analyzed using WinRHIZO^™^ software (Regent Instruments Inc., Canada). Remaining fine and coarse roots were dried and weighed as above. Leaf area ratio (total leaf area per total plant dry mass, m^2^ g^-1^; LAR) and specific leaf area (leaf area per leaf dry mass, m^2^ g^-1^; SLA), and leaf mass area (leaf dry mass per leaf area, g m^-2^; LMA) were calculated using the leaf data for each plant. Specific root length of fine roots (root length per dry mass, m g^-1^; SRL_FineRts_) and total root mass fraction (RMF, total root mass per total plant weight) were calculated using the fine root length and root biomass data from each plant [31, 62].

At the end of the recovery phase, all plants were also destructively sampled for measurement of leaf area and above- and below-ground biomass partitioning as above.

### Field study

A field trial was conducted during the dry season of 2015 to evaluate the performance of 29 eggplant genotypes including Mara, PHL 4841, PHL 2778 and PHL 2789 at the experimental farm of the Institute of Plant Breeding, University of the Philippines Los Baños. The data and findings of the other 25 genotypes are presented in another manuscript. Plants were fertilized with a total of 170-70-180 kg N, P, K ha^-1^. Fertilizers were applied at planting, 3 weeks after transplanting (WAT), 6 WAT and during the fruiting stage. Three-week-old- seedlings of each genotype were transplanted in 4 row plots, 4 m in length, with 0.75 m distance between row and 0.5 m distance between plants.

The trial was laid out in a split plot design. Watering treatment (moisture stress and well-watered treatments as the main plot while the accessions/varieties were assigned to the smallest plots. The trial had 4 replications, with 4 row- plots measuring 4 meters in length, and 0.75 meter row distance.

Water stress was imposed by withholding irrigation among the water stress treatment plots at 5 WAT while the control plots were irrigated weekly through furrow irrigation. Water stress was imposed for two weeks and was terminated with rainfall occurrence after the 2^nd^ week of irrigation witholding. After the heavy rain, stressed plants were allowed to recover with equal irrigation to control plots.

Six plant samples per genotype were collected for biomass partitioning, initial harvest, and root traits determination after termination of water stress treatment. Plant samples were partitioned into roots, stem, leaves and fruits. Leaves were cleaned and measured for leaf area using Li-3100C leaf area meter (Li-COR, Lincoln, Nebraska, USA). The roots were cleaned and washed free of soil particles prior to air drying for 2–3 days and eventually oven dried for 48 hours at 60°C together with above ground biomass. Dried samples were weighed on a top loading balance.

Two harvests were obtained during water stress phase while two harvests were collected at recovery. Mature fruits were harvested from 5 plants from inner and center rows and weighed immediately for total fresh weight.

### Data analysis

Distributions of trait residuals were assessed for non-normality and transformed as needed. Traits for each ANOVA were examined for unequal variances within each treatment factor combination and the validity of differences between means of unequal variances were checked using Welch’s ANOVA and, when necessary, non-parametric Pair-wise Comparisons using Wilcoxon’s method. To assess the effect of water stress on *S*. *melongena*, all traits were analyzed with a Least-squares means test by Water stress treatment (control vs. water stress), with Genotype as a random effect (REML method). All analyses were performed in JMP v.13 except for VarClus cluster analysis (SAS) and PCAs (R).

To assess the differential response of the four *S*. *melongena* genotypes to water stress, all traits were analyzed with a one-way ANOVA with Genotype as the main effect. Finally, to determine and visualize which traits dominate the *S*. *melongena* water stress response and to understand trait partitioning among the four genotypes, we performed a Principal Component Analysis (PCA) in R using the prcomp() function in ggplot2. We chose the following nine variables for the PCA based on cluster analysis and a priori hypotheses of which traits might contribute to different drought-adaptive strategies: stem dry mass, fine root dry mass, A_n_, transpiration rate, F_v_/F_m_, Ψ_L_, SRL, SLA, and RMF.

To assess the effect of recovery post-water stress on *S*. *melongena*, all trait data measured after the recovery phase were analyzed with a Least-squares means test by Water stress treatment (control vs. water stress) with Genotype as a random effect (REML method). To assess the persistence of genotype-specific trait values for traits that displayed significant differences among genotypes during the water stress phase, trait data measured directly following the recovery phase for plants under water stress was analyzed with a one-way ANOVA with Genotype as the main effect.

## Results

### Response of *S*. *melongena* to water stress (prior to recovery watering)

Among the 25 traits measured, all but four were negatively impacted by the water stress treatment (LSM analysis, p< 0.0031, Table 1). The four traits which did not significantly change in response to water stress were all fine root traits: basal fine root mass, total fine root volume, fine root tissue density, and specific fine root length. Importantly, water stress significantly limited growth rate and photosynthetic activity relative to well-watered controls among all four genotypes. The mean maximum A_n_ rate of well-watered plants was 22.19 ± 1.28 μmol CO_2_ m^-2^ s^-1^ whereas plants under water stress attained maximum A_n_ rates of only 5.92 ± 1.16 μmol CO_2_ m^-2^ s^-1^ (LSM analysis, p < 0.0001, Table 1). Aboveground biomass (leaf dry weight, shoot biomass, and stem dry mass) was significantly reduced in plants under water stress as stem dry mass was 11.47 ± 1.26 g for well-watered plants compared to the much lower mass of plants under water stress, 7.40 ± 1.26 g (LSM analysis, p < 0.0001, S1 Table in S1 File). Belowground traits also followed this trend, with coarse and fine root biomass being significantly reduced in plants under water stress. Notably, however, plants under water stress had a significantly greater root:shoot ratio and RMF than the controls (15 and 20% greater; LSM analysis, p < 0.0002, Table 1). Morphological changes in SLA contributed to the reduction in leaf area for plants under water stress (SLA = 0.013 ± 0.0006 m^2^ g^-1^) relative to well-watered plants (SLA = 0.018 ± 0.0006 m^2^ g^-1^, LSM analysis, p < 0.0001, Table 1). Finally, hydraulic status of plants under water stress also declined regardless of genotype relative to control plants; leaf water potential became much more negative (-2.299 ± 0.119 vs. -0.814 ± 0.119 MPa, p<0.0001).

**Table 1:** Standard least squares analysis of physiological traits with Genotype as a random effect (REML method) during water stress phase.

Trait	DF	F-ratio	Prob > F	Mean of Control Plants	Std Error	Mean of plants under water stress	Std Error
**Leaf water potential (MPa)**	1	958.264	< 0.0001	-0.814	0.119	-2.299	0.119
**Total leaf area (cm^2^)**	1	770.056	< .0001	2899.885	88.519	839.575	88.519
**Fv/Fm**	1	51.771	< .0001	0.825	0.004	0.794	0.004
**Photosynthetic Rate (μmoles CO_2_ m^-2^ s^-1^)**	1	106.223	< .0001	22.195	1.277	5.918	1.163
**Stomatal Conductance (mmoles H_2_O m^-2^ s^-1^)**	1	127.134	< .0001	0.377	0.022	0.044	0.020
**Transpiration Rate (μmoles H_2_O m^-2^ s^-1^)**	1	148.120	< .0001	5.391	0.259	0.948	0.229
**Water Use Efficiency (g dry weight g^-1^ H_2_O)**	1	18.791	0.0001	4.281	0.282	6.352	0.233
**Leaf Dry Weight (Green, g)**	1	385.915	< .0001	16.101	0.783	6.606	0.783
**Leaf Dry Weight (Senesced, g)**	1	49.528	< .0001	1.548	0.585	4.294	0.585
**Stem Dry Weight (g)**	1	94.472	< .0001	11.474	1.255	7.360	1.255
**Shoot dry weight (g)**	1	184.146	< .0001	29.123	2.400	18.259	2.400
**Leaf Area Ratio (cm^2^ g^-1^)**	1	218.023	< .0001	75.600	4.662	33.981	4.686
**Specific Leaf Area (cm^2^ g^-1^)**	1	119.135	< .0001	0.018	0.001	0.013	0.001
**Basal fine root mass (g)**	1	0.274	0.6041	1.993	0.424	1.764	0.424
**Total fine root mass (g)**	1	29.593	< .0001	4.923	0.696	3.879	0.696
**Coarse root mass (g)**	1	18.292	0.0001	5.172	0.977	4.175	0.978
**Total root mass (g)**	1	29.008	< .0001	10.096	1.646	8.079	1.647
**Root:Shoot Ratio**	1	30.353	< .0001	0.343	0.030	0.429	0.030
**Root Mass Fraction**	1	27.273	< .0002	0.254	0.015	0.298	0.015
**Total Fine root length (cm)**	1	13.266	0.0009	72166.132	6926.092	54854.872	6971.446
**Total fine root surface area (m^2^)**	1	10.182	0.0031	969.050	98.947	771.316	100.022
**Total fine root volume (m^3^)**	1	2.512	0.1225	0.209	0.022	0.168	0.022
**Specific fine root length (m g^-1^)**	1	1.230	0.2755	164.807	20.533	140.817	21.162
**Fine root tissue density (g m^-3^)**	1	0.210	0.6499	26.743	2.325	28.810	2.540
**Fine root length: Leaf area ratio**	1	35.008	< .0001	25.174	6.229	71.750	6.368

Standard error is pooled on the verified assumption that treatment variances are statistically equal. Each trait is comprised of 40 observations with the exception of fine root measurements (only 19 samples in the deficit treatment) and gas exchange parameters (only 16 samples in the control treatment). Plants were harvested at 7 weeks post germination following a 2-week water stress treatment.

### Responses among genotypes of *S*. *melongena* to water stress (prior to recovery)

**Fig 1:**
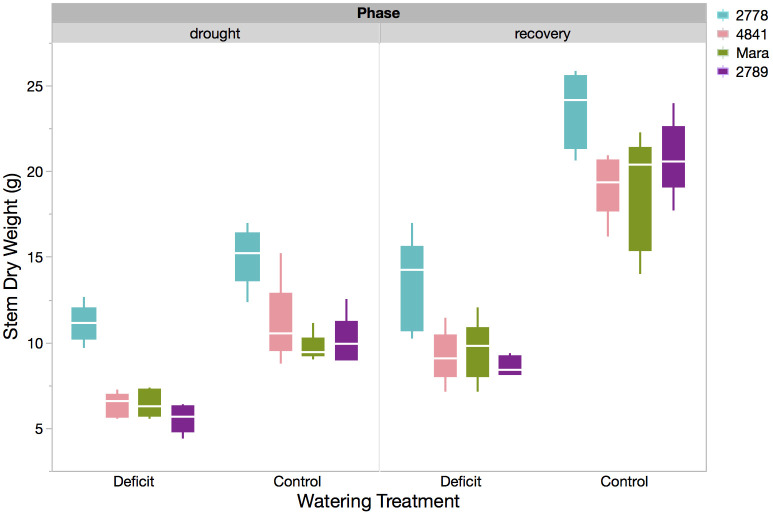
Box plot of stem dry weight (g) for each genotype in both the water stress and control treatments after re-watering recovery (right panel). The solid box contains the inner two quartiles, whereas whisker lines indicate 1.5 x IQR (3^rd^ quartile minus the 1^st^ quartile) from the box. The white horizontal line is the mean.

**Fig 2:**
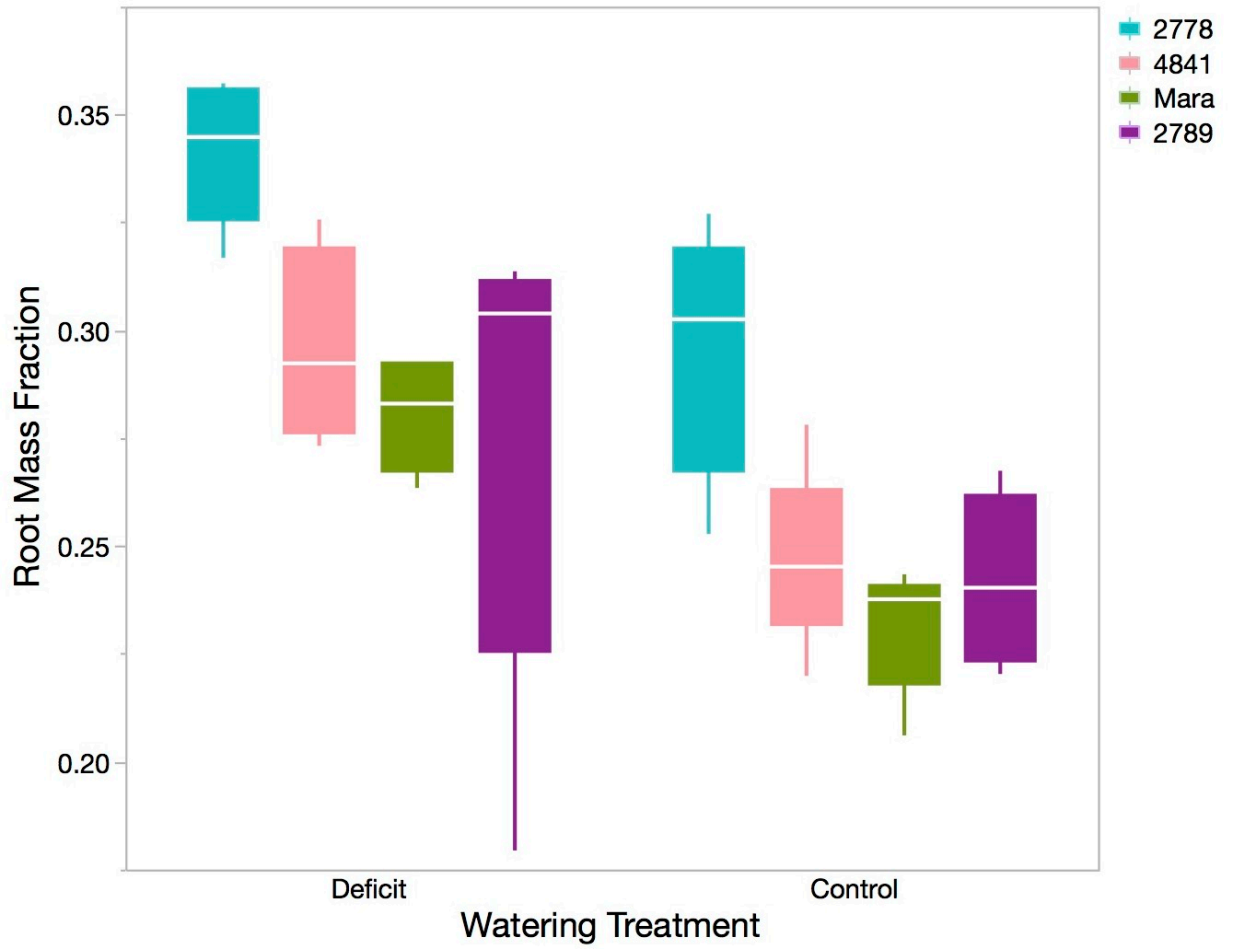
Box plot of root mass fraction for each genotype in both the water stress and control treatments. The solid box contains the inner two quartiles, whereas whisker lines indicate 1.5 x IQR (3^rd^ quartile minus the 1^st^ quartile) from the box. The white horizontal line is the mean.

For many traits, there were clear varietal differences. Across all organs, PHL 2778 had the highest biomass at the end of the water stress period, while PHL 2789 tended to have the lowest. Mara and PHL 4841 were generally intermediate in growth and biomass. Variation in stem biomass among genotypes typified the pattern in which PHL 2778 accumulated higher biomass (11.11 ± 0.39 g) than Mara (6.43 ± 0.39 g), PHL 4841 (6.34 ± 0.39 g), and PHL 2789 (5.56 ± 0.39 g, ANOVA, p< 0.0001, Table 2, Fig 1, left panel). Notably, the genotype with the highest total biomass also allocated the most to root biomass: PHL 2778 had the highest RMF, PHL 4841 intermediate, and Mara and PHL 2789 the lowest (ANOVA, p = 0.001; Table 2, Fig 2).

**Table 2:** ANOVA of physiological traits of plants under water stress in response to Genotype during the water stress phase.

Trait	DF	F-ratio	Prob > F	Mean PHL 2778	Std Error	Mean PHL 4841	Std Error	Mean Mara	Std Error	Mean PHL 2789	Std Error
**Leaf water potential (MPa)**	3	25.627	< .0001	-2.468	0.061	-2.652	0.061	-2.064	0.061	-2.012	0.061
**Total leaf area (cm^2^)**	3	0.643	0.5984	900.684	97.633	738.690	97.633	902.406	97.633	816.518	97.633
**Fv/Fm**	3	2.295	0.1168	0.779	0.007	0.798	0.007	0.797	0.007	0.803	0.007
**Photosynthetic Rate (μmoles CO_2_ m^-2^ s^-1^)**	3	0.503	0.6856	5.866	1.231	6.329	1.231	4.726	1.231	6.750	1.231
**Stomatal Conductance (mmoles H_2_O m^-2^ s^-1^)**	3	1.266	0.3195	0.045	0.009	0.053	0.009	0.030	0.009	0.047	0.009
**Transpiration Rate (μmoles H_2_O m^-2^ s^-1^)**	3	0.846	0.4890	0.981	0.190	1.082	0.190	0.694	0.190	1.036	0.190
**Water Use Efficiency (g dry weight g^-1^ H_2_O)**	3	0.713	0.5584	6.154	0.688	5.687	0.688	7.065	0.688	6.501	0.688
**Leaf Dry Weight (Green, g)**	3	2.373	0.1086	7.831	0.645	6.150	0.645	6.914	0.645	5.528	0.645
**Leaf Dry Weight (Senesced, g)**	3	8.321	0.0015	6.345	0.603	4.487	0.603	4.254	0.603	2.092	0.603
**Stem Dry Weight (g)**	3	41.323	< .0001	11.110	0.394	6.338	0.394	6.426	0.394	5.564	0.394
**Shoot dry weight (g)**	3	46.549	< .0001	25.285	0.744	16.975	0.744	17.593	0.744	13.184	0.744
**Leaf Area Ratio (cm^2^ g^-1^)**	3	4.392	0.0208	23.469	4.476	29.405	5.004	37.592	4.476	45.107	4.476
**Specific Leaf Area (cm^2^ g^-1^)**	3	4.528	0.0176	0.011	0.001	0.012	0.001	0.013	0.001	0.015	0.001
**Basal fine root mass (g)**	3	1.619	0.2244	2.757	0.540	1.675	0.540	1.400	0.540	1.226	0.540
**Total fine root mass (g)**	3	26.120	< .0001	5.837	0.263	3.642	0.263	3.155	0.263	2.882	0.263
**Coarse root mass (g)**	3	63.819	< .0001	7.263	0.270	3.496	0.302	3.716	0.270	2.201	0.270
**Total root mass (g)**	3	60.953	< .0001	13.099	0.445	7.229	0.498	6.871	0.445	5.083	0.445
**Root:Shoot Ratio**	3	6.812	0.0010	0.318	0.011	0.269	0.012	0.256	0.011	0.259	0.011
**Root Mass Fraction**	3	5.253	0.0112	0.520	0.027	0.422	0.030	0.390	0.027	0.387	0.027
**Total Fine root length (cm)**	3	2.520	0.0974	70648.910	7025.651	48781.992	7854.916	45296.921	7025.651	53182.001	7025.651
**Total fine root surface area (m^2^)**	3	4.376	0.0227	1039.389	85.527	664.221	95.622	644.564	95.622	722.082	85.527
**Total fine root volume (m^3^)**	3	0.612	0.6186	0.209	0.039	0.130	0.044	0.162	0.044	0.167	0.039
**Specific fine root length (m g^-1^)**	3	1.621	0.2293	118.640	21.869	134.660	24.450	127.132	24.450	181.515	21.869
**Fine root tissue density (g m^-3^)**	3	0.813	0.5076	29.915	5.705	35.993	6.378	22.924	6.378	25.741	5.705
**Fine root length: Leaf area ratio**	3	0.788	0.5190	86.638	16.019	79.925	17.910	53.992	16.019	67.696	16.019

Standard error is pooled on the verified assumption that treatment variances are statistically equal. Each trait is comprised of 40 observations with the exception of fine root measurements (9 samples of Mara) and gas exchange parameters (9 samples per genotype). Plants were harvested at 7 weeks post germination following a 2-week water stress treatment.

**Fig 3:**
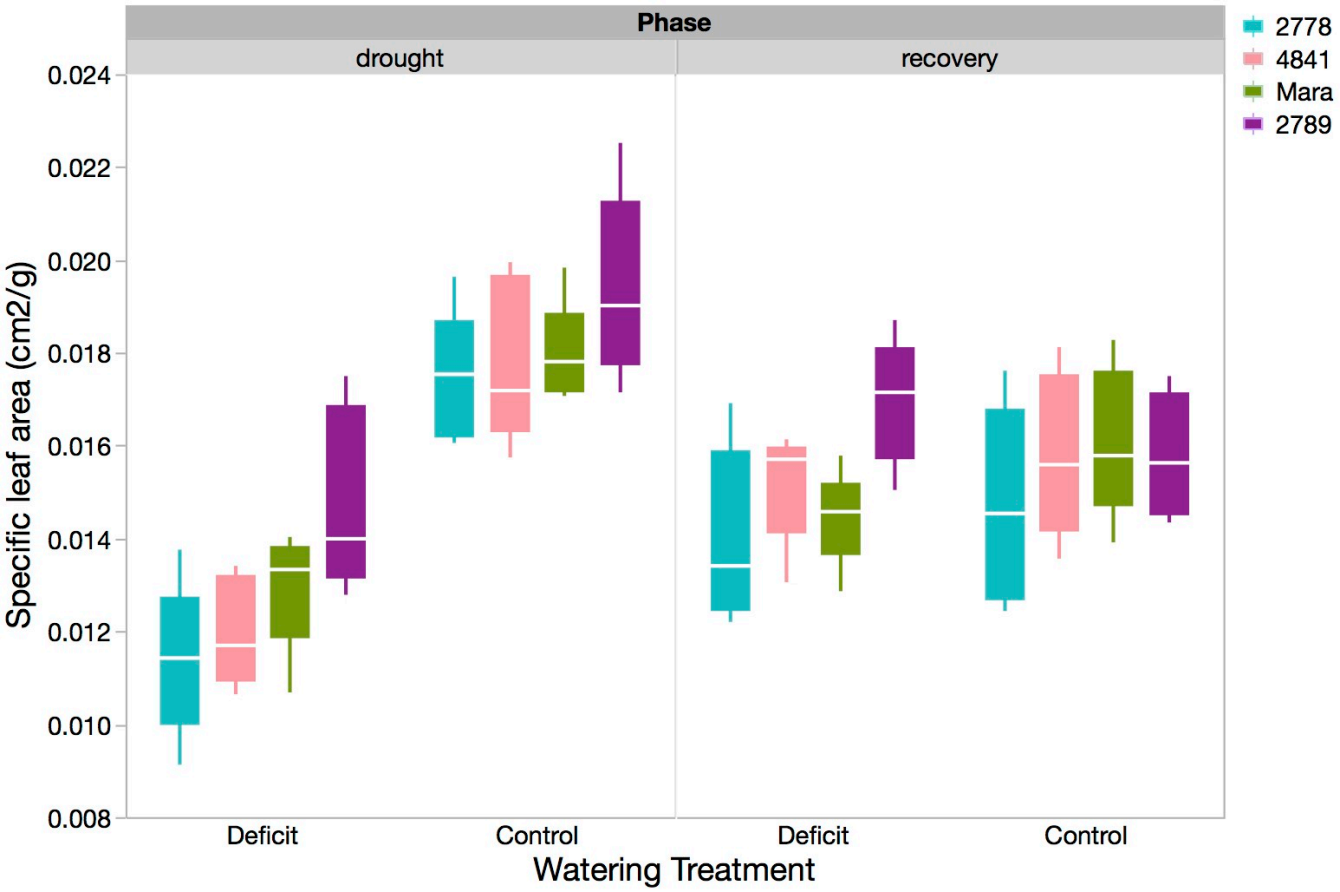
Box plot of Specific leaf area (cm^2^/g) for each genotype in both the water stress and control treatments immediately following 2 weeks of drought (left panel) and following 1 week of re-watering recovery (right panel). The solid box contains the inner two quartiles, whereas whisker lines indicate 1.5 x IQR (3^rd^ quartile minus the 1^st^ quartile) from the box. The white horizontal line is the mean.

**Fig 4:**
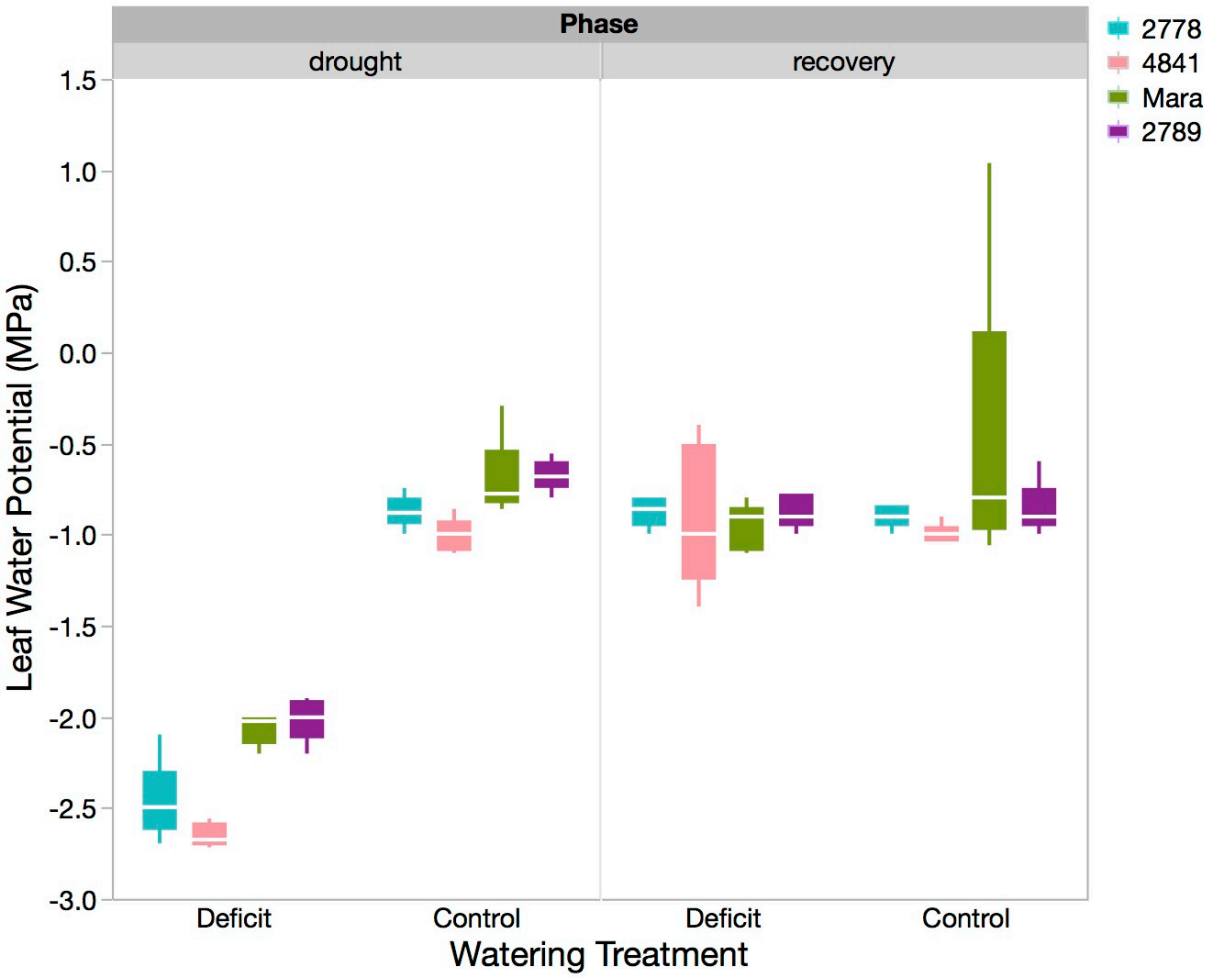
Box plot of leaf water potential (MPa) for each genotype in both the water stress and control treatments after re-watering recovery (right panel). The solid box contains the inner two quartiles, whereas whisker lines indicate 1.5 x IQR (3^rd^ quartile minus the 1^st^ quartile) from the box. The white horizontal line is the mean.

**Fig 5:**
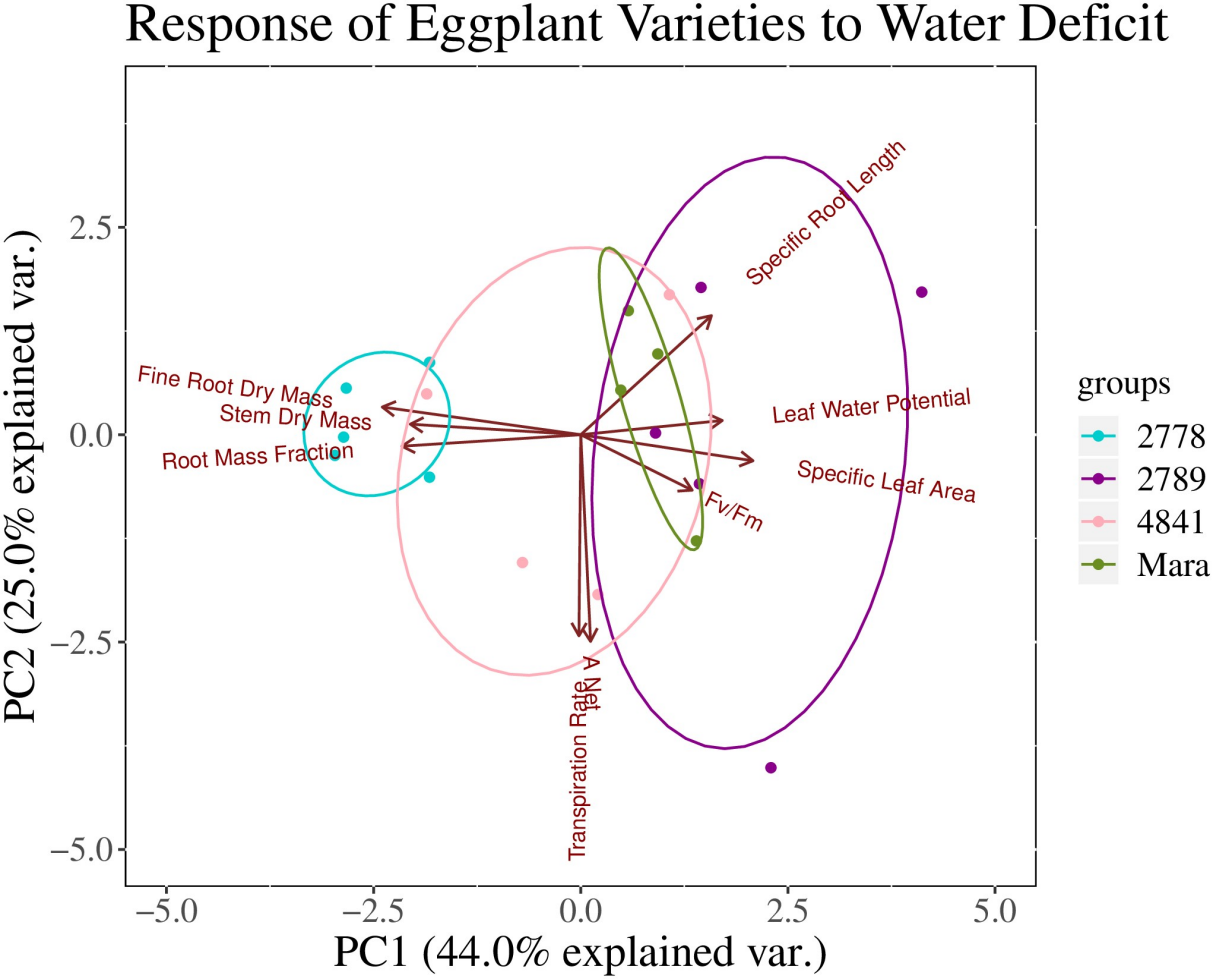
Principal component analysis of the water stressed samples prior to recovery watering. Loading arrows indicate the magnitude and direction of trait variation (9 traits) underlying the sample distribution. Ellipses indicate groupings by genotype.

An opposite trend was observed with leaf morphology and plant hydraulic status, where the smaller plants of PHL 2789 had higher leaf area per unit dry weight (SLA), potentially due to better hydraulic status, supporting higher leaf area expansion than the larger plants of PHL 2778 (Table 2, Fig 3). PHL 2789 displayed the lowest hydraulic stress as indicated by a higher Ψ_L_ under water stress (-2.01 ± 0.06 MPa), followed by Mara (-2.07 ± 0.06 MPa), PHL 2778 (-2.47 ± 0.06 MPa) and PHL 4841 (-2.65 ± 0.06 MPa) (ANOVA, p< 0.0001, Table 2, Fig 4, left panel). PCA illustrates the broader differences between genotypes and their strategies in response to water stress (Fig 5). Greater biomass and proportional root allocation were observed in PHL 2278 and associated with more negative Ψ_L_, reduced electron transport through photosystem II (low F_v_/F_m_ values), smaller, thicker leaves (low SLA) and shorter SRL_FineRts_. On the other hand, PHL 2789 and Mara maintained better hydraulic status and electron transport, with larger, thinner leaves, but produced less root and shoot biomass.

Other traits that demonstrated significant varietal differences were root:shoot ratio, LMA, senesced leaf dry mass, LAR, total shoot mass, total root mass, fine root mass, coarse root mass, and total fine root surface area (ANOVA, Table 2).

The remaining traits responded to water stress on a species level with no significant differences among genotypes in the water stress response (ANOVA, Table 2). These traits included all gas exchange/photosynthetic traits (A_n_, F_v_/F_m_ ratio, *g*_s_, transpiration, WUE_i_) as well as some plant morphological and biomass allocation traits (e.g., total leaf area, green leaf mass, basal fine root mass, SRL_FineRts_, total fine root volume, and total FRL:LA ratio).

### Re-watering and recovery in water stressed *S*. *melongena*

Recovery via re-watering after water stress restored the following traits impacted by water stress back to values observed in control plants: physiological traits (F_v_/F_m_ and Ψ_L_, Fig 4, right panel), biometric traits (basal fine root mass and fine root tissue density), and allometric traits (LAR, RMF, SRL_FineRts_, FRL:LA ratio, and SLA, Fig 3, right panel). Full recovery for these traits was inferred from the lack of significant difference between mean values of control (well-watered) and re-watered plants that underwent water stress or, in the case of F_v_/F_m_, increase of trait values in re-watered plants beyond that of control plants (LSM analysis, Table 3).

**Table 3:** Standard least squares analysis of physiological traits with variety as a random effect (REML method) after recovery phase.

Trait	DF	F-ratio	Prob > F	Total recovery?	Mean of Control Plants	Std Error	Mean of plants under water stress	Std Error
**Leaf water potential (MPa)**	1	0.591	0.4471	yes	-0.814	0.072	-0.900	0.072
**Total leaf area (cm^2^)**	1	101.143	< .0001	no	3425.336	176.413	1954.980	176.413
**Fv/Fm**	1	3.986	0.0537	yes	0.817	0.001	0.822	0.001
**Leaf Dry Weight (Green, g)**	1	208.950	< .0001	no	21.941	1.410	13.083	1.410
**Leaf Dry Weight (Senesced, g)**	1	16.136	0.0003	no	2.147	0.452	4.290	0.452
**Stem Dry Weight (g)**	1	232.169	< .0001	no	20.587	1.096	10.168	1.096
**Shoot dry weight (g)**	1	391.393	< .0001	no	44.675	2.481	27.540	2.481
**Leaf Area Ratio (cm^2^ g^-1^)**	1	2.270	0.1409	yes	56.679	3.261	52.215	3.261
**Specific Leaf Area (cm^2^ g^-1^)**	1	0.749	0.3927	yes	0.016	0.000	0.015	0.000
**Basal fine root mass (g)**	1	1.069	0.3083	yes	1.181	0.168	1.012	0.168
**Total fine root mass (g)**	1	54.391	< .0001	no	7.077	0.790	5.114	0.790
**Coarse root mass (g)**	1	102.061	< .0001	no	9.245	0.868	5.618	0.868
**Total root mass (g)**	1	126.148	< .0001	no	16.322	1.634	10.732	1.634
**Root:Shoot Ratio**	1	2.523	0.1212	yes	0.364	0.026	0.388	0.026
**Total Fine root length (m)**	1	11.997	0.0015	no	8714.401	649.100	5778.892	664.533
**Total fine root surface area (m^2^)**	1	13.013	0.0010	no	1398.161	108.069	963.301	109.943
**Total fine root volume (m^3^)**	1	9.471	0.0041	no	0.297	0.025	0.186	0.026
**Specific fine root length (m g^-1^)**	1	1.165	0.2880	yes	12.230	0.889	10.919	0.912
**Fine root tissue density (g m^-3^)**	1	1.452	0.2364	yes	28.997	3.436	36.525	3.583
**Fine root length: Leaf area ratio**	1	0.768	0.3870	yes	2.663	0.319	3.000	0.325
**Root mass fraction**	1	2.452	0.1264	yes	0.266	0.014	0.278	0.014

Standard error is pooled on the verified assumption that treatment variances are statistically equal. Each trait is comprised of 40 observations with the exception of fine root measurements (only 19 samples in the deficit treatment). Plants were harvested at 8 weeks post germination following one week of recovery watering. “Total recovery” is considered to have occurred when the mean trait value of control plants is no longer significantly different from the mean trait value of plants under water stress.

The following traits did not attain full recovery to control plant levels within the week after re-watering, as evidenced by their significant differences from control plants after recovery treatment (LSM analysis, Table 2): total shoot mass, stem mass, total leaf area, green leaf mass, senesced leaf mass, fine root mass, coarse root mass, total root mass, total fine root length, fine root surface area, and fine root volume (Fig 2, right panel). These traits were all a function of plant growth rate, which certainly increased in during recovery but not enough to match the final biomass values of the consistently watered control plants.

### Elimination of genotypic differences by recovery watering

Most traits that exhibited water stress-induced genotypic differences maintained these significant differences after re-watering (ANOVA, S1 Table in S1 File). As examples, stem dry mass remained lowest in PHL 2789 (8.62 ± 0.82 g) and highest in PHL 2778 (13.37 ± 0.82 g, ANOVA, p = 0.0033, Fig 2, right panel); and SLA remained highest in PHL 2789 (0.0170 ± 0.0006 m^2^ g^-1^) and lowest in PHL 2778 (0.0140 ± 0.0006 m^2^ g^-1^, ANOVA, p = 0.0237, Fig 3, right panel). Other traits include LAR, senesced leaf mass, total shoot mass, fine root mass, coarse root mass, total root mass, total fine root mass, RMF, and root:shoot ratio.

For two traits, however, recovery after re-watering eliminated the genotype-specific differences observed during water stress: neither leaf water potential (Fig 4, right panel) nor total fine root surface area displayed significant differences between genotypes after re-watering (ANOVA, S1 Table in S1 File).

### Linking greenhouse observations to field-grown plants

Stem dry mass, total shoot dry mass, root dry mass, and LMA significantly correlated with fresh fruit mass in water stressed eggplant in field-grown trials (Pearson Correlation Coefficient, S2 Table in S1 File). Using the strongest relationship (root dry mass, R^2^ = 0.30, p = 0.0053) to project fresh fruit production from the greenhouse-grown plants in this study, we found that water stressed plants would have produced, at best, 83% of the fresh fruit yield of irrigated plants. PHL 2778 and Mara were projected to produce more fruit biomass under drought relative to their irrigated control (246.58 g/plant or 83% of irrigated controls and 154.26 g/plant or 77% of irrigated controls, respectively) than PHL 4841 and PHL 2789 (producing only 173.22 g/plant or 75% of irrigated controls and 135.09 g/plant or 67% of irrigated controls, respectively).

## Discussion

The four *S*. *melongena* genotypes in this study appeared to use two different strategies in response to water stress during the vegetative phase. The first strategy is that of being a “water spender”, similar to the “early vigor” and “drought avoidance” models in agronomy and ecology [11, 12, 16, 25]. One such genotype was PHL 2778, which produced large, fast growing, plants with extensive root systems that quickly accessed water but experienced rapidly declining photosynthetic function and water status as the water supply was depleted. On the other hand, genotypes Mara and PH: 2789 were “water savers”, displaying hallmarks of classic drought tolerance [12, 16]. Mara and PHL 2789 were slow growing, smaller plants that maintained safer water use thresholds by using their available soil water more conservatively (Fig 5). Field data confirmed that larger *S*. *melongena* plants under water stress tended to have lower SLA (the inverse of specific leaf weight, which has a significant relationship with stem dry mass, S2 Table in S1 File), which could be an adaptive trait to reduce transpirational water loss. Interestingly, gas exchange parameters, and, thus, WUE_i_, did not differ among the genotypes, indicating that while smaller, the leaves of PHL 2778 did not have more sensitive g_s_ thresholds or lower A_n_. Ultimately, genotypic differences in the ontogeny of root expansion and depletion of pot water, as indicated by RMF and Ψ_L_, were the main traits underpinning the productivity of different genotypes under water stress.

In general, increased RMF is a common allometric adjustment to drought, and may be driven by biomechanical differences in root turgor vs. shoot turgor under water deficit [63]. Root traits of *S*. *melongena* found here upheld previous observations that increased rooting investment largely results in increased productivity during drought (Table 2, Fig 2) [31, 64]. In oats, drought tolerant genotypes had increased root length, root surface area and length of fine root hairs [40, 65]. Additionally, deep root systems have been found to be associated with drought tolerance in common bean and with hydraulic efficiency thereby contributing to the fitness of monocots under drought [27, 66]. Plant strategies of substantial biomass allocation to root structures are generally expected to generate a tradeoff of decreased whole plant biomass accumulation. Conversely, a strategy of greater proportional aboveground biomass would increase photosynthetic carbon assimilation and thus increase biomass production [67]. The lack of such a tradeoff here could be due to slow-growing genotypes having additional sensitivities under water stress that limited their growth, such as limited cell expansion or starch accumulation (since WUE_i_ did not differ among genotypes).

Remarkably, increased rooting investment in *S*. *melongena* genotypes did not translate to superior hydraulic status. In fact, results were the opposite: Ψ_L_ was more negative and LAR was smaller in PHL 2778 and PHL 4841, the genotypes with greater RMF, root mass, and total biomass (Table 2, Fig 5). These trait patterns may have been a result of plant confinement in pots, given that greenhouse-grown plants possessed finite pot space and limited irrigation was supplied daily. A more extensive root system may have allowed PHL 2778 and PHL 4841 to use up the water more quickly in their pots leading to more negative Ψ_L_. It is possible that in the field, the deeper roots PHL 2778 and PHL 4841 would access additional water from deep soils and maintain Ψ_L_ and leaf area closer to those of the smaller, more hydraulically conservative genotypes (Mara and PHL 2789).

When drought is episodic, plant response to re-watering and plant performance under water stress are equally important for achieving acceptable yields [34]. This study identifies traits that remain impaired beyond the water stress exposure period and traits that recovered quickly in response to re-watering in *S*. *melongena*. After recovery via re-watering, genotypic differences were largely maintained in biomass-related traits (total and specific LA, dry stem weight, and root biomass). Physiological traits having to do with photosynthesis, hydraulic status, and labile biomass such as leaves and fine roots, however, lost their initial water stress-induced genotypic differences (F_v_/F_m_, RMF, fine root length, fine root SA, and Ψ_L_) (Fig 4). These traits showed a high degree of short-term (1 week) recovery potential, suggesting the activation of a broad suite of key recovery traits such as membrane and photosystem repair, embolism recovery, and fine root flushing [8, 31, 68, 69]. Given early enough drought and a longer growing season remaining for *S*. *melongena* after resumption of adequate precipitation in the Philippines, there may be potential for more substantial biomass recovery across genotypes beyond that observed in this study.

Given that PHL 2778 achieved significantly greater biomass than the other genotypes under water stress in this study, and that biomass (of the leaves, shoot, and/or root) significantly correlated with fresh fruit production, it is clear that drought avoidance is the most promising strategy for moderate drought stress in eggplant. Breeding for rapid growth and, perhaps, early flowering will likely increase yields in *S*. *melongena* whether or not the plants experience drought in during the growing season. The other three genotypes, particularly Mara and PHL 2789, fall into the category of plants whose drought tolerance comes at a cost to biomass and productivity [16, 22, 70, 71]. They are thus less desirable for integrating into current crop genotypes. It should be noted, however, that these less productive, drought tolerant genotypes may outperform large drought avoiders like PHL 2778 when grown under more severe or repetitive drought, as it has been observed in maize [22]. Under current model of projected episodic drought in the Philippines, however, fast-growing genotypes such as PHL 2778 may provide the greatest agricultural benefit. The strategy of early high root investment and fast biomass growth compensates for rapidly declining hydraulic status to produce greater fruit yield, with strong potential for additional yield recovery after re-watering.

In conclusion, the identification of key trait responses to water stress will be useful for guiding selection of parental lines for varietal improvement, providing a clear desired trait outcome during the hybrid screening process. In this case, evaluation of water stress strategies among a set of demonstrated drought tolerant genotypes showed a range of whole-plant strategies, from small but tolerant water-savers to large, productive, but profligate water spenders. Given that these trait responses were evaluated in a greenhouse study, it will be important to verify the consistency of these strategies under field conditions where linkages to yield can also be confirmed, a necessary step to validate them as targets for breeding efforts [5].

## Supporting information

S1 File Contains supporting S1 and S2 Tables. (PDF)
