# Supplementary material for: Plant strategies for maximizing growth during water stress and subsequent recovery in Solanum melongena L. (eggplant)
Source: PLoS One. 2021 Sep 1;16(9):e0256342. doi: 10.1371/journal.pone.0256342 (PMC8409672; doi:10.1371/journal.pone.0256342)
Supplement: S1 File — (PDF) [file pone.0256342.s001.pdf]

Supplementary Materials

TITLE: Plant strategies for maximizing growth during water stress and subsequent recovery in *Solanum melongena* L. (eggplant)

AUTHORS: Evelyn F. Delfin[1]*, Sarah T. Drobnitch[2], and Louise H. Comas[3]

[1]Institute of Plant Breeding, University of the Philippines, Los Baños College, Laguna 4031.

[2]Soil and Crop Department, Colorado State University, Fort Collins, CO 80526.

[3]United States Department of Agriculture, Agricultural Research Service, 2150 Centre Avenue, Fort Collins, CO 80526.

*efdelfin1@up.edu.ph

Table S1. ANOVA of physiological traits of deficit plants in response to Genotype after the recovery phase
Showing only traits with significant variety differences during drought.

| Trait | DF | F-ratio | Prob > F | Variety differences still present? | Mean 2778 | Std Error | Mean 4841 | Std Error | Mean Mara | Std Error | Mean 2789 | Std Error |
|---|---|---|---|---|---|---|---|---|---|---|---|---|
| Leaf water potential | 3 | 0.1631 | 0.9197 | no | -0.872 | 0.098 | -0.900 | 0.098 | -0.956 | 0.098 | -0.872 | 0.098 |
| Leaf Dry Weight (Senesced) | 3 | 4.8094 | 0.0142 | yes | 5.630 | 0.663 | 4.892 | 0.663 | 4.390 | 0.663 | 2.246 | 0.663 |
| Stem Dry Weight | 3 | 6.9735 | 0.0033 | yes | 13.368 | 0.820 | 9.182 | 0.820 | 9.504 | 0.820 | 8.616 | 0.820 |
| Shoot dry weight | 3 | 25.5673 | <.0001 | yes | 34.434 | 1.082 | 26.409 | 1.082 | 28.150 | 1.082 | 21.165 | 1.082 |
| Leaf Area Ratio | 3 | 6.21 | 0.0053 | yes | 43.190 | 2.822 | 50.581 | 2.822 | 55.611 | 2.822 | 59.476 | 2.822 |
| Specific Leaf Area | 3 | 4.1428 | 0.0237 | yes | 0.014 | 0.001 | 0.015 | 0.001 | 0.014 | 0.001 | 0.017 | 0.001 |
| Specific Leaf Weight | 3 | 3.6976 | 0.034 | yes | 72.406 | 2.929 | 66.318 | 2.929 | 69.465 | 2.929 | 59.287 | 2.929 |
| Total fine root mass | 3 | 16.1868 | <.0001 | yes | 7.417 | 0.400 | 5.030 | 0.400 | 4.077 | 0.400 | 3.933 | 0.400 |
| Coarse root mass | 3 | 11.0706 | 0.0004 | yes | 7.859 | 0.489 | 5.696 | 0.489 | 4.803 | 0.489 | 4.114 | 0.489 |
| Total root mass | 3 | 25.8502 | <.0001 | yes | 15.276 | 0.635 | 10.726 | 0.635 | 8.880 | 0.635 | 8.047 | 0.635 |
| Root:Shoot Ratio | 3 | 5.1071 | 0.0114 | yes | 0.447 | 0.024 | 0.404 | 0.024 | 0.318 | 0.024 | 0.381 | 0.024 |
| Total fine root surface area | 3 | 1.1302 | 0.3685 | no | 1138.330 | 153.409 | 991.908 | 153.409 | 744.096 | 153.409 | 982.281 | 171.516 |
| Root mass ratio | 3 | 5.3912 | 0.0093 | yes | 0.308 | 0.012 | 0.287 | 0.012 | 0.241 | 0.012 | 0.275 | 0.012 |

Table S2. Correlation among biomass, leaf traits and total fruit yield of 29 varieties of eggplant grown under water-stress followed by recovery watering in the field in 2015. Light green, $p < 0.05$. Dark green, $p < 0.001$. SLDW – Senesced leaves dry weight; SDW – Stem dry weight; VB – Vegetative biomass, TDMY- Total dry matter yield, % GLDW – Percent green leaves dry weight; %SLDW – percent senesced leaves dry weight, % SDW – percent stem dry weight; %RDW – Percent root dry weight dry weight; TLA- Total leaf area; SLW – Specific leaf weight; TFW – Total fruit weight.

| | SLDW | SDW | RDW | VB | TDMY | %GLDW | % SLDW | % SDW | % RDW | TLA | SLW | TFW |
|---|---|---|---|---|---|---|---|---|---|---|---|---|
| **GLDW** | -0.26 | 0.66 | 0.71 | 0.73 | 0.72 | 0.88 | -0.81 | 0.09 | 0.09 | 1 | 0.65 | 0.5 |
| **SLDW** | | 0.24 | 0.17 | 0.38 | 0.34 | -0.51 | 0.53 | -0.09 | -0.27 | -0.26 | 0.05 | -0.13 |
| **SDW** | | | 0.86 | 0.95 | 0.92 | 0.32 | -0.56 | 0.48 | 0.04 | 0.66 | 0.55 | 0.44 |
| **RDW** | | | | 0.87 | 0.9 | 0.39 | -0.63 | 0.19 | 0.37 | 0.71 | 0.52 | 0.55 |
| **VB** | | | | | 0.96 | 0.39 | -0.5 | 0.27 | -0.03 | 0.73 | 0.62 | 0.43 |
| **TDMY** | | | | | | 0.36 | -0.57 | 0.13 | -0.02 | 0.72 | 0.6 | 0.53 |
| **% GLDW** | | | | | | | -0.77 | 0.04 | 0.1 | 0.88 | 0.64 | 0.37 |
| **% SLDW** | | | | | | | | -0.2 | -0.3 | -0.8 | -0.56 | -0.6 |
| **% SDW** | | | | | | | | | 0.21 | 0.09 | 0.11 | 0 |
| **% RDW** | | | | | | | | | | 0.1 | -0.15 | 0.19 |
| **TLA** | | | | | | | | | | | 0.65 | 0.5 |
| **SLW** | | | | | | | | | | | | 0.44 |
